# A Fiber-Based SPR Aptasensor for the In Vitro Detection of Inflammation Biomarkers

**DOI:** 10.3390/mi13071036

**Published:** 2022-06-29

**Authors:** Yu Hua, Ridong Wang, Dachao Li

**Affiliations:** State Key Laboratory of Precision Measuring Technology and Instruments, Tianjin University, Tianjin 300072, China; huayu_19@tju.edu.cn (Y.H.); dchli@tju.edu.cn (D.L.)

**Keywords:** fiber-based surface plasmon resonance, DNA aptamer, surface density distribution, C-reactive protein, cardiac troponin I

## Abstract

It is widely accepted that the abnormal concentrations of different inflammation biomarkers can be used for the early diagnosis of cardiovascular disease (CVD). Currently, many reported strategies, which require extra report tags or bulky detection equipment, are not portable enough for onsite inflammation biomarker detection. In this work, a fiber-based surface plasmon resonance (SPR) biosensor decorated with DNA aptamers, which were specific to two typical inflammation biomarkers, C-reactive protein (CRP) and cardiac troponin I (cTn-I), was developed. By optimizing the surface concentration of the DNA aptamer, the proposed sensor could achieve a limit of detection (LOD) of 1.7 nM (0.204 μg/mL) and 2.5 nM (57.5 ng/mL) to CRP and cTn-I, respectively. Additionally, this biosensor could also be used to detect other biomarkers by immobilizing corresponding specific DNA aptamers. Integrated with a miniaturized spectral analysis device, the proposed sensor could be applied for constructing a portable instrument to provide the point of care testing (POCT) for CVD patients.

## 1. Introduction

With the fast aging of the world population, cardiovascular disease (CVD) is becoming one of the leading causes of death [1,2]. The imaging methods, including ultrasonic wave [3] and computed tomography [4], may not be suitable for the early diagnosis of CVD because of their hysteresis while the CVD-related inflammation biomarker (such as C-reactive protein (CRP) [5,6], cardiac troponin I (cTn-I) [7], brain natriuretic peptide (BNP) [8], and interleukins [9]) detection can be a promising method to diagnose CVD at an early stage because the levels of certain inflammation biomarkers can indicate the risk of CVD sharply. Many biosensors for CVD-related inflammatory biomarker detection, including electrochemical sensors [10], fluorescent spectrometry sensors [11], and colorimetric sensors [12], have been developed. Although these strategies could achieve high sensitivity and a low detection limit, an inevitable limitation is that chemically modified tags converting the concentration of the inflammation biomarkers to a readable signal are required in these biosensors, which complicates the detection operation. Moreover, an inappropriate store condition may lead to the inactivation of these modification tags, which affects the sensor’s reliability. 

Surface plasmon resonance (SPR) biosensors, which are label-free, have advantages in the early detection of CVD [13]. However, the commercialized prism-coupled SPR biosensor is not suitable for the POCT device due to its bulky size [14]. The fiber-based SPR biosensor, which employs an optical fiber to transmit the surface plasmon excitation and reflection light [15], largely decreases the sensor’s size and manufactory cost. Furthermore, the utilization of the integrated LED light source [16] and the microspectrograph enables the elimination of bulky testing equipment in the fiber-based SPR biosensors, which can promote the application of portable SPR biosensors for an onsite clinical CVD diagnosis.

In order to realize the specific detection of inflammation biomarkers, it is necessary to modify specific biometric receivers on the fiber-based SPR sensor’s surface. Antibodies are widely used as biometric receivers to achieve high sensitivity and low detection limits [17,18]. However, the drawbacks, including pricey synthesis cost [19], fragile bioactivity [20], and fixed affinity [21], restrict the application of antibodies in fiber-based SPR sensors. DNA aptamers have advantages [22], including more economical production costs, adjustable affinity, and a simpler modification process, which may help to break through the limitation of antibodies. Additionally, compared to antibodies, most DNA aptamers have the same chemical groups [23]. Therefore, it is feasible to design similar modification and detection processes for different DNA aptamers, which can primarily increase the efficiency of proposing aptasensors for the different CVD marker detection. 

In this work, a fiber-based SPR biosensor for detecting two typical inflammatory biomarkers, CRP and cTn-I, was exhibited. The corresponding DNA aptamers with optimized concentrations were immobilized onto the surface of the sensor to realize highly specific detection of CRP and cTn-I. The limit of detections of 1.7 nM (0.204 μg/mL) and 2.5 nM (57.5 ng/mL) to CRP and cTn-I were achieved, respectively. Furthermore, the specificity and the affinity of the DNA aptamers could be adjusted by changing the DNA sequence to realize a flexible detection of different biomarkers. Additionally, this biosensor could also be integrated with a microfluidic system and a miniaturized spectrum detector to construct a POCT device for onsite CVD-related inflammation biomarker detection.

## 2. Materials and Methods

### 2.1. Materials and Reagents

The chemicals used were obtained from the following sources: bovine serum albumin (BSA) was purchased from Amresco Co. (Cleveland, OH, USA). NaCl, CaCl_2_, glycine, tris base, sodium dodecyl sulfate polyacrylamide (SDS), and ethylenediaminetetraacetic acid (EDTA) were purchased from Meryer Co., LTD., Shanghai, China. Hexamethylhexanol (MCH) and trichloroethyl phosphate (TCEP) were purchased from Maclin Co., LTD., Shanghai, China. Unless specifically mentioned, all reagents used were of the analytical grade or higher. The solution was prepared using the diethylpyrocarbonate (DEPC)-treated ultrapure water and filtered by the 0.22 μm polytetrafluoroethylene microfiltration membrane. Buffers used in the experiment were as follows:

− Binding buffer: 20 mM tris base, 1 mM EDTA, 1 mM TCEP, pH = 8.0
− Detection buffer: 25 mM tris base, 192 mM glycine, 0.05% Tween 20, 0.1 w/t% BSA, 2 mM CaCl_2_, 100 mM NaCl, pH = 8.0
− Washing buffer: 20 mM tris base, 1 mM EDTA, 0.001% SDS, pH = 8.0

The DNA aptamer specific to CRP and cTn-I was synthesized by Sangon Co., LTD., Shanghai, China. The sequence of the aptamer is listed in Table 1 [24,25]. The ploy T spacer, which was five base pairs in length, was added to the 3’ end of the aptamer to decrease the steric hindrance effect from the surface assembly membrane and to help the aptamer stay upright on the sensor surface. Additionally, the thiol linker was added to the 3′ end to help to immobilize the aptamer firmly onto the biosensor’s surface. The human recombinant CRP and the human recombinant cTn-I (produced from the Escherichia coli) for the evaluation of the biosensor were purchased from Biolab Co., LTD., Beijing, China, and Sangon Co., LTD., Shanghai, China, respectively.

The multimode fiber (core diameter: 625 μm) and the standard SMA 905 interface were purchased from Xinrui Co., LTD., Shenzhen, China. The Cr (99.9%) and Au (99.9%) utilized as the target materials for evaporation were purchased from ZhongNuo Advanced material (Beijing) Technology Co., LTD., Beijing, China. The glass tubes for packaging the SPR biosensor were purchased from Tianyanggu Co., LTD., Shenzhen, China. The polyethylene tube utilized as the flow chamber of the sensor was purchased from Asone Co., LTD., Shanghai, China. 

### 2.2. Fiber-Based Biosensor Fabrication and System Set-Up

**Figure 1:**
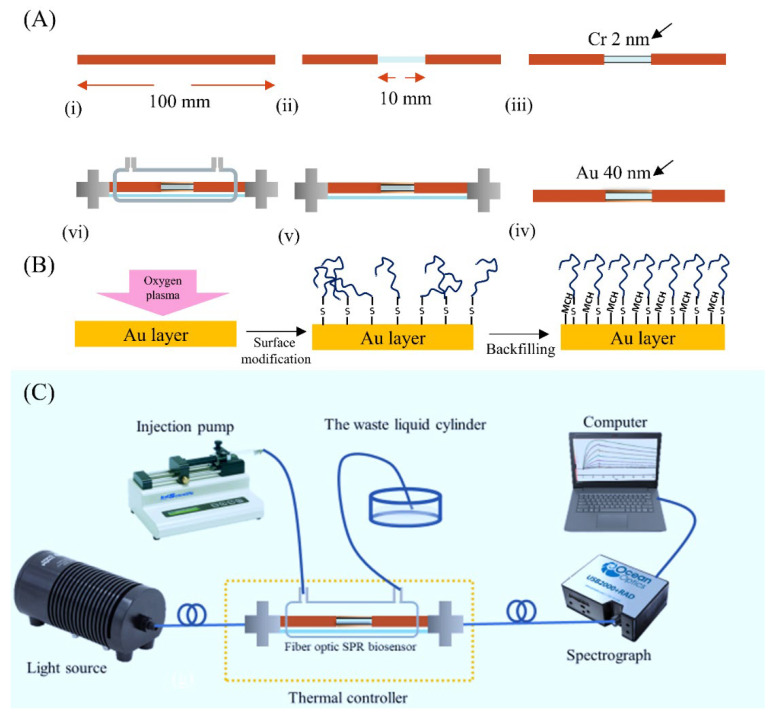
(**A**) The manufactory process of the biosensor: (i) fiber preparation; (ii) cladding layer exfoliation; (iii) chromium deposition; (iv) gold deposition; (v) sensor packaging; (vi) flow chamber packaging (**B**) The surface modification of the biosensor. (**C**) The scheme of the whole detection system.

Figure 1A shows the fabrication process of the fiber-based SPR biosensor. Firstly, a 100 mm-length optical fiber with a 10 mm-length sensing area was prepared. The sensing area was scraped to wipe off the cladding layer. Then, the pretreated fiber was incubated in an acetone solution for 15 min and then washed three times using distilled water. Next, the fiber was dried out and embedded in a special fixture for a thermal evaporative coating. According to our previous research [26,27], a 2 nm-thick chromium film and a 40 nm-thick gold film as the optimization were deposited sequentially on the surface of the fiber. After that, the coated fiber was heated at the temperature of 80 °C for 2 h and then annealed to room temperature tardily to release the residual stress of coated layers. Next, the fiber surface was hydrophobically treated under oxygen plasma (Dongxin High-tech Automation Equipment Co., LTD., Shenzhen, China) for 30 s. Finally, in order to preserve the sensor’s integrity and biocompatibility, the fiber was encapsulated with a glass tube and standard SMA 905 interfaces and enclosed with the polyethylene microchamber. 

Figure 1B shows the DNA aptamer modification on the sensor surface. First, the thiol-modified DNA aptamer specific to the certain inflammation biomarker was treated with 1 mM TCEP for 1 h at room temperature to selectively reduce the occurrence of DNA dimers, which was harmful to the aptamer immobilization process. Then, the solution containing the DNA aptamer was loaded onto the sensing area of the fiber-based SPR and was incubated at 4 °C overnight. After that, the sensor was washed using a PBS buffer and distilled water, respectively. To backfill the remaining binding sites on the fiber surface, the sensor was later incubated in 2 mM MCH at room temperature for 90 min. At last, the sensor was washed repeatedly with distilled water and stored under 4 °C for further use.

Figure 1C shows the whole biosensing system, including a wide band light source (HL2000, Ocean Optic, Dunedin, FL, USA), a fiber-based SPR biosensor, a micro spectrograph (USB 2000, Ocean Optic, Dunedin, FL, USA), a computer for data processing, an injection pump (KD Scientific, Holliston, MA, USA), and a cylinder for the waste liquid. The fiber-based SPR biosensor was connected with the light source and spectrograph using the standard SMA interface to realize the excitation of the SPR and the detection of the SPR shift, respectively. The thermal controller we proposed was utilized to decrease the temperature fluctuation around the SPR biosensor. The sensor was treated with the washing buffer for 5 min to recover its surface and then treated with the detection buffer for 1 min to wash off the residual SDS. The flow rate during the process was held at 15 μL/min by the injection pump. 

The proposed SPR biosensor’s surface was characterized by the scanning electron microscope (SEM, Nanosem 430, FEI, USA) and the X-ray photoelectron spectroscopy (XPS, ESCALAB-250 Xi Thermo, Altrincham, UK). The surface roughness of the glass sheet was characterized by the atomic force microscope (AFM, Bruker, Germany). The infiltration characteristics were measured by the contact angle measuring system (OSA60, New Boundary Co., Ningbo, China).

## 3. Results and Discussion

### 3.1. The Characterization of the Sensing Areas

**Figure 2:**
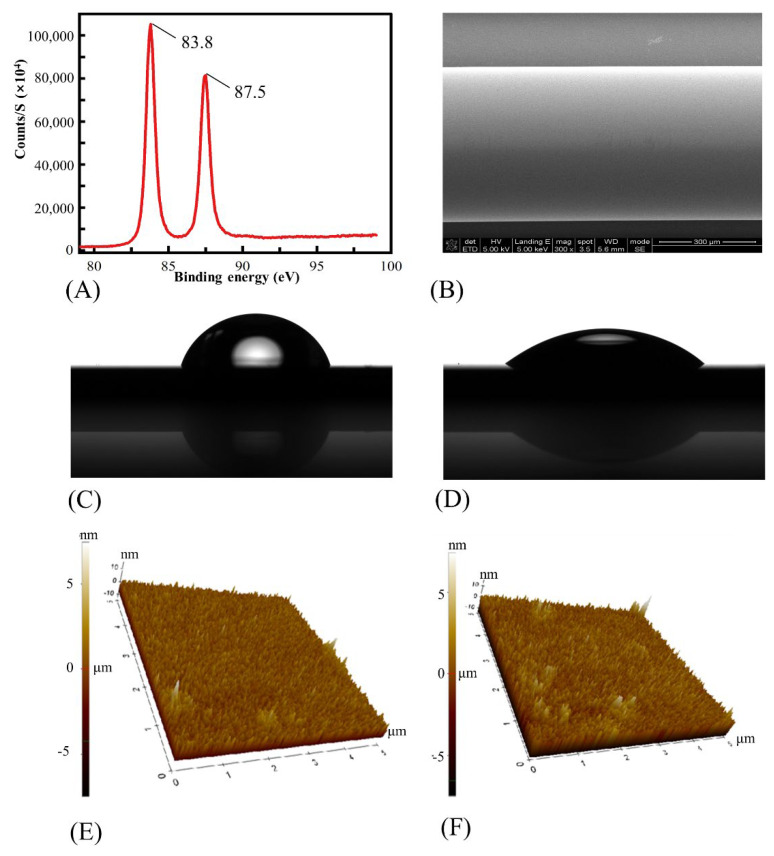
(**A**) The XPS evaluation of the sensing area of the biosensor. (**B**) The SEM evaluation of the sensing area of the biosensor. (**C**,**D**) The infiltration characteristics of the glass sheet before and after the oxygen plasma treatment. (**E**,**F**) The surface morphology of the glass sheet with and without oxygen plasma treatment.

The surface morphology of the proposed fiber-based SPR biosensor was characterized. As shown in Figure 2A, two peaks at the binding energy of 83.8 eV and 87.5 eV were observed, which indicated that the Au layer was deposited onto the fiber successfully. Figure 2B shows the sensing area of the sensor, which also confirmed that the smooth metal layers were successfully coated onto the sensing area.

In order to let the DNA aptamer absorb onto the coated fiber much more efficiently, it was necessary to improve the hydrophilicity of the fiber surface. Here, oxygen plasma treatment was used to produce hydrophilic polar bonds without serious damage to the fiber surface. In addition, compared with the commonly used surface treatment based on liquid chemistry (like the piranha solution), the oxygen plasma treatment was friendly to the following modification process due to zero chemical pollution. The infiltration characteristics with and without oxygen plasma treatment were shown in Figure 2C,D. Here, the surface contact angles of the ultrapure water on the gold surface of the glass sheet were used to evaluate the surface hydrophilia. The contact angle of the gold surface decreased from 75° to 36°, showing the significant hydrophilia improvement of the gold surface after the surface plasma treatment. To evaluate the damage to the sensing area caused by the oxygen plasma, Figure 2E,F showed the surface morphology of the gold surface with (Figure 2E) and without (Figure 2F) oxygen plasma treatment. It could be seen that the roughness of the gold surface was changed (from 1.64 nm to 1.85 nm). However, our actual test results showed that the increase in surface roughness did not significantly affect the basic performance of our sensor (the results were not shown here).

### 3.2. Basic Performance Testing of the SPR Biosensor

**Figure 3:**
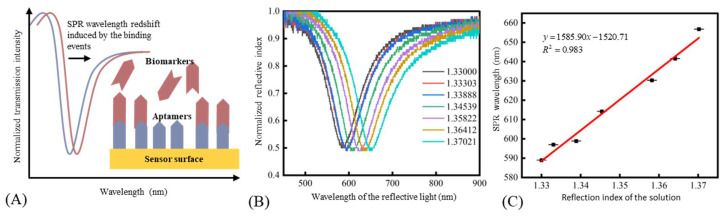
(**A**) The measurement principle of SPR biosensors. (**B**) The normalized spectrum of the sensor under solutions with different RIs. (**C**) The relationship between the SPR wavelength and the refractive index of the solutions.

The detection principle of SPR biosensing technology was to distinguish the refractive index (RI) change near the sensor surface. As shown in Figure 3A, the binding events of aptamers and biomarkers would lead to the RI increase near the sensor surface, which caused the redshift of the SPR wavelength. Therefore, it was necessary to evaluate the proposed SPR biosensor’s response to the RI change before the chemical modification process. The RI of the solution was adjusted by using glycerine solutions with different concentrations. Here, the temperature around the sensor was fixed at 20 °C to avoid the effect of temperature variation on the measured RI [28]. As shown in Figure 3B, the resonance peak shifted with the increase of RI. Figure 3C shows a good linear relationship between the resonance wavelength and RI, and the sensitivity was 1585.90 nm/RIU in a RI range of 1.33–1.37.

### 3.3. CRP and cTn-I Detection 

**Figure 4:**
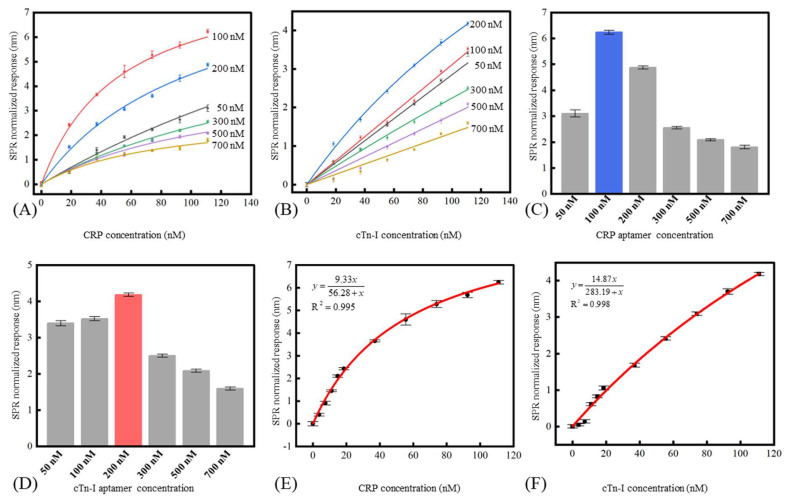
The sensor’s response to the CRP (**A**) and cTn-I (**B**) under different concentration aptamer modification. The responses of the sensors were modified with different concentration aptamers under the 111 nM CRP (**C**) and cTn-I (**D**). The response and the detection limit of the biosensor to CRP (**E**) and cTn-I (**F**).

Since the aptamer’s surface loading density had a prominent impact on the SPR biosensor’s response [29], optimizing the DNA aptamer’s surface density was necessary. Here, the aptamer’s surface loading density was controlled by incubating the CRP and cTn-I aptamers with different concentrations (50 nM, 100 nM, 200 nM, 300 nM, 500 nM, 700 nM) under the same incubating time. As shown in Figure 4A,B, the sensors with low surface density of the aptamer (like the sensor modified with 50 nM CRP aptamer and cTn-I aptamer) had the relative low response and high saturation concentration. The sensors with medium surface density of the aptamer (like the sensor modified with 100 nM and 200 nM aptamers for CRP and cTn-I detection) had the higher response. However, their saturation concentration decreased because of the steric hindrance from the analyte. As for the sensors with a high surface density of the aptamer (including the sensors modified with 300 nM aptamers or higher), both the sensor response and the saturation concentration diminished because of the negative effect induced by the crowded aptamers’ surface distribution. Figure 4C,D showed the responses of the different SPR biosensors under 111 nM CRP and 111 nM cTn-I. Theoretically, a higher surface density of aptamer led to a higher response of the sensor because more binding sites were offered in the limited area [30]. However, the response of the SPR biosensor increased when the modified CRP aptamer and cTn-I aptamer concentration increased from 50 nM to 100 nM and 50 nM to 200 nM, respectively, and then the response began to decrease with the concentrations of the CRP aptamer and cTn-I aptamer increasing from 100 nM to 700 nM and 200 nM to 700 nM, respectively. On the one hand, the decrease in the sensor response could be attributed to the steric hindrance effect [31,32,33]. Denser aptamer’s surface distribution referred to the shorter distance between the neighboring aptamers. Therefore, the existed complex of aptamers and biotargets on the sensor surface were more likely to overshadow the rest free aptamers around, which prevented the extra biotargets from binding onto the sensor. On the other hand, high aptamer coverage density would suppress the aptamer’s affinity. It was acknowledged that aptamers needed to self-fold to certain hydrophilic, pocket-shaped structures [34,35], which was necessary before binding with biotargets [36]. However, the ultra-crowded aptamer’s distribution might cause two issues, which could block the aptamers from folding into the expected constructures. The first issue regarded electrostatic interference. The aptamers were immobilized closely, so they might fail to fold appropriately because of the electrostatic force from each other [37]. The second regarded thermodynamic interference. Due to the self-complementary nature of the individual aptamer sequences, the aptamers would like to hybridize with neighboring aptamers [38] rather than self-fold. Therefore, it was essential to control the aptamer’s density to weaken the adverse effect of the lack of binding sites, the steric hindrance, the electrostatic force, and the random cross-hybridization to the sensor’s response. 

**Figure 5:**
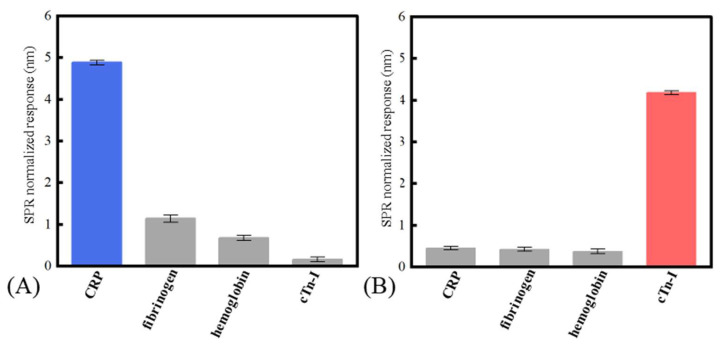
The selectivity of the biosensor: (**A**) the sensor modified with the CRP aptamer; (**B**) the sensor modified with the cTn-I aptamer.

**Figure 6:**
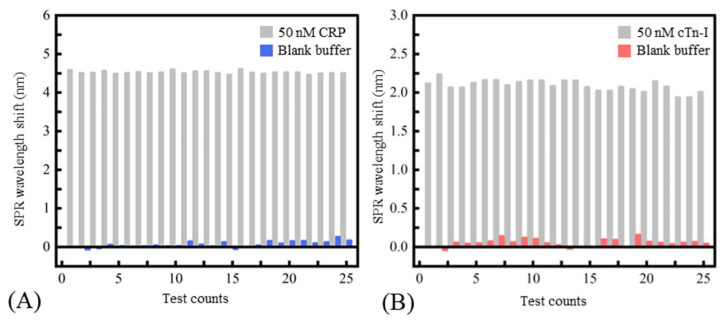
The repeatability of the biosensor: (**A**) the sensor modified with the CRP aptamer; (**B**) the sensor modified with the cTn-I aptamer.

According to the results from Figure 4C,D, concentrations of 100 nM for CRP aptamer and 200 nM for cTn-I were used for the fiber-based SPR biosensor. Figure 4E,F showed the sensor response under different CRP and cTn-I concentrations. The sensitivity of the SPR biosensor could be calculated using the following formula:

(1) $$S=\frac{\Delta \lambda }{\Delta C}$$

where S, ΔC, and Δλ were the sensitivity of the SPR biosensor, the concentration changes of the CRP or cTn-I, and the corresponding wavelength shift of the SPR peak, respectively. The LOD of the SPR biosensor could be calculated through the following formula:

(2) $$\mathrm{LOD}=\frac{3\times \sigma_{\mathrm{background}}}{S_{\mathrm{background}}}$$

where $\sigma_{\mathrm{background}}$ referred to the standard deviation of the wavelength shift of the SPR biosensor. $S_{\mathrm{background}}$ referred to the biosensor’s sensitivity near the blank sample (obtained by calculating the slope of the fitted curve near the blank sample). According to the experiment results, the LOD of the SPR biosensor to the CRP and cTn-I was 1.7 nM (0.204 μg/mL) and 2.5 nM (57.5 ng/mL), respectively. In addition, solutions of 100 nM CRP, 100 nM hemoglobin, 100 nM fibrinogen, and 100 nM cTn-I were used to evaluate the specificity of the fiber-based SPR sensor. Figure 5A,B showed the specificity of the SPR sensor modified with CRP aptamer and the cTn-I aptamer, respectively. From the results, the nonspecific binding responses between the sensor and the disturbed biomolecules were less than 20% of the specific binding between the sensor and the certain inflammation biomarkers, indicating that the aptamer chosen had a prominent affinity to the corresponding biomarkers and the conspicuous stain resistance of the SAM made of MCH. Figure 6A,B shows the sensor’s repeatability. The sensor’s responses to the 50 nM of CRP and 50 nM of cTn-I were measured repeatedly. It could be seen that the relative standard deviation values of the sensor’s response to CRP and cTn-I were only 2.15% and 4.72%, respectively, indicating that the proposed biosensor had a good ability to reuse because of the robust surface modification protocols.

## 4. Conclusions

In this paper, a label-free, fiber-based SPR aptasensor for CVD-related inflammation biomarker detection was developed. Two typical CVD-related inflammation biomarkers (CRP and cTn-I) for early diagnosis of CVD were detected successfully by immobilizing specific DNA aptamers. The results showed that the SPR sensor had a LOD of 1.7 nM (0.204 μg/mL) and 2.5 nM (57.5 ng/mL) to CRP and cTn-I, respectively. Moreover, the results of the selectivity and repeatability test preliminarily demonstrated the sensor’s capability for biosensing in complex matrices. Due to the generalizability of our method, more CVD-related biomarkers could be measured through our proposed biosensor.

It should be noted that the aptamer’s surface loading density urgently affected the sensor’s response, which could be attributed to the lack of the binding sites, the steric hindrance, the electromagnetism, and the thermodynamic effect. Although the surface density of the aptamer could be adjusted by the modified concentration, it was inevitable that the aptamer partially crowded the sensing area because of the molecular dynamics in the solution [39]. Another limitation was that the detection window might not meet the biomedical detection requirements because the sensing performance of our proposed biosensor was largely determined by the aptamer’s affinity and the molecule weight of the biotarget. Moreover, it was necessary to further evaluate the practicality of the sensor under the real blood serum. Currently, we are trying to introduce the programmable DNA frameworks to the SPR biosensor to control the surface distribution of the aptamer more precisely and to realize some novel biosensing functions under the nanoscale. In addition, we are trying to introduce the mixture of the aptamers with different affinities to adjust the sensor’s detection window and to modify two-dimensional material and noble metal nanoparticles for enhancing the sensor’s sensitivity. Furthermore, we are attempting to combine the proposed biosensor with a microfluidic system and a miniaturized spectrum detector to promote the practical application of our proposed biosensor as an integrated POCT device for CVD diagnosis. In future research, we will test the optimized sensor under the human blood serum to provide a further demonstration of its practicality.

## Figures and Tables

**Table 1:** The list of the DNA aptamers for different inflammation markers.

Aptamer Type	The Sequence (5′ to 3′)
CRP	GGCAGGAAGACAAACACGATGGGGGGGTATGATTTGATGTGGTTGTGCATGATCGTGGTCTGTGGTGCTGTTTTT
cTn-I	CGAAGGGGATTCGAGGGGTGATTGCGTGCTCCATTTGGTGTTTTT

## Data Availability

Not applicable.
